# High-Flow Congenital Arteriovenous Malformation of the Right Deltoid Muscle in a Pediatric Patient: A Case Report

**DOI:** 10.7759/cureus.80580

**Published:** 2025-03-14

**Authors:** Mohammad Zaid, Suhaila Ahmed, Avdullah Qafani, Ayman Al-Sibaie, Alaa E Nwilati, Michael G Shahoud, Mamoun Shafaamri, Masoud Shafiei, Asmaa Haj Husin, Deena M Al Qedrah, Mohammad S Faghih

**Affiliations:** 1 Urology, Dubai Health, Dubai, ARE; 2 Vascular Surgery, Dubai Health, Dubai, ARE; 3 Radiology, Dubai Health, Dubai, ARE; 4 Medicine, Mohammed Bin Rashid University of Medicine and Health Sciences, Dubai, ARE

**Keywords:** arteriovenous malformations, embolization, pediatric, surgical resection, vascular anomalies

## Abstract

Congenital arteriovenous malformations (AVMs) are rare vascular anomalies involving abnormal connections between arteries and veins, bypassing the capillary bed. High-flow AVMs may lead to complications such as pain, ulceration, and functional impairment. We report a case of a six-year-old girl with a high-flow AVM in the right deltoid muscle, initially diagnosed via Doppler ultrasound and confirmed by MRI. The patient presented with a progressively enlarging, painless swelling of the right shoulder. Management included super-selective embolization, followed by surgical resection due to symptom recurrence and persistent vascular shunting. Postoperatively, the patient experienced improved shoulder mobility and pain reduction. The treatment plan involved a staged approach, with initial embolization to reduce blood flow, followed by surgical excision to prevent recurrence. The surgical resection was performed soon after the second embolization. Postoperative care included pain management and physiotherapy for optimal recovery. This case emphasizes the importance of early diagnosis, multimodal intervention, and long-term follow-up in pediatric AVMs. Future studies should focus on recurrence predictors, optimal timing for surgical resection post-embolization, and the role of genetic factors in AVM development.

## Introduction

Congenital arteriovenous malformations (AVMs) are rare vascular anomalies present from birth, characterized by abnormal direct connections between arteries and veins, bypassing the capillary bed and resulting in high-flow lesions [1]. While AVMs commonly occur in the brain and extremities, intramuscular AVMs, particularly in the deltoid muscle, are exceedingly rare and present unique diagnostic and management challenges [2]. These lesions can cause progressive pain, swelling, functional impairment, and, in severe cases, ulceration or bleeding.

The deltoid muscle is an uncommon location for AVMs, making this case significant. AVMs have been reported in muscles such as the temporalis muscle without affecting its function [3]. High-flow AVMs within muscle tissue pose a greater risk of recurrence due to their complex vascular network and potential for collateral vessel formation [4]. Understanding their pathophysiology is crucial for optimal management. Recent research has identified genetic mutations, such as those in the EPHB4 and RAS/MAPK pathways, as contributing factors to AVM formation, providing new insights into targeted treatment approaches [5].

The management of high-flow intramuscular AVMs typically involves a multidisciplinary approach, combining transcatheter embolization to reduce arterial inflow, followed by surgical resection to prevent recurrence [6]. The timing of surgery is critical, with most experts recommending excision within 2 to 8 weeks post-embolization to minimize revascularization [7]. However, complete resection remains challenging due to deep-seated components and persistent feeders [8].

This case report highlights the rare occurrence of a high-flow deltoid AVM in a pediatric patient, emphasizing the importance of early diagnosis, multidisciplinary treatment strategies, and long-term follow-up to prevent recurrence. By presenting this case, we aim to contribute to the growing body of literature on the optimal management of intramuscular AVMs and the evolving role of genetic and targeted therapies in their treatment.

## Case presentation

A six-year-old female presented with swelling in her right deltoid region following trauma. She had accidentally struck her right shoulder against a door key 10 days prior while playing. Four days after the incident, her father noticed swelling in the area. The swelling was painless and non-tender, with no associated symptoms such as active bleeding or discomfort. There was no limitation of right shoulder movement, and the parents were uncertain if the mass was present before the injury. The swelling developed gradually over a few days, and the child reported no pain or movement limitations, with no systemic symptoms like fever, fatigue, or bruising. The patient had no significant past medical history, known allergies, or family history of bleeding disorders or clotting factor deficiencies.

**Figure 1:**
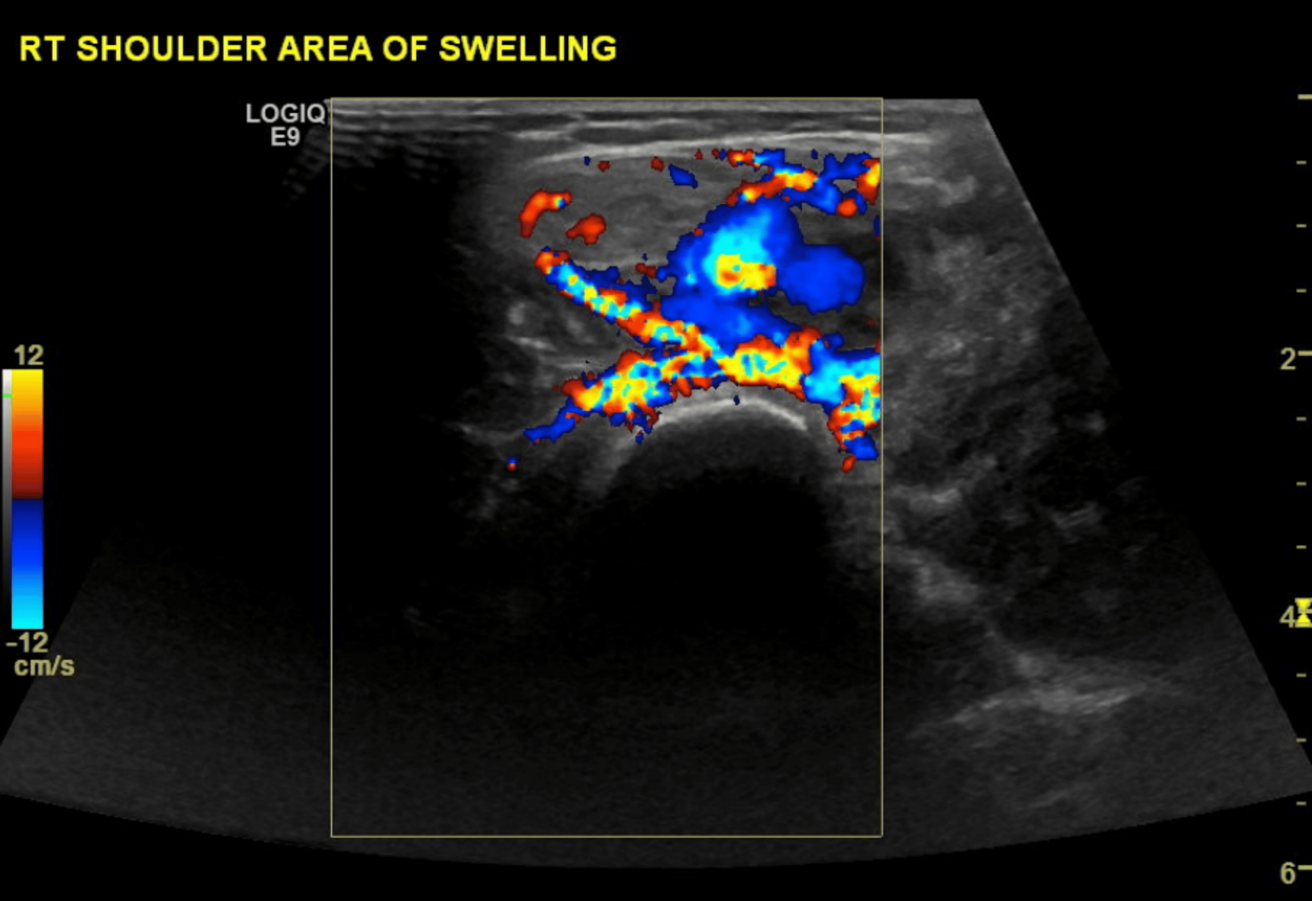
Ultrasound of right deltoid muscle High-flow arteriovenous malformation (AVM) within the right deltoid muscle, with no evidence of hematoma or fluid collection. Doppler ultrasound showed high diastolic venous flow and arterial flow consistent with an AVM.

On examination, the patient weighed 28.3 kg, had a height of 130 cm, and a BMI of 16.75 kg/m². A 5 × 7 cm soft, compressible, and pulsatile swelling was noted over the lateral-anterior aspect of the right deltoid region. The mass was mobile over the muscle, warm to the touch, and non-tender. Radial and ulnar pulses were intact, with no distal neurovascular compromise or associated lymphadenopathy. An ultrasound was performed, and its results are shown in Figure 1.

**Figure 2:**
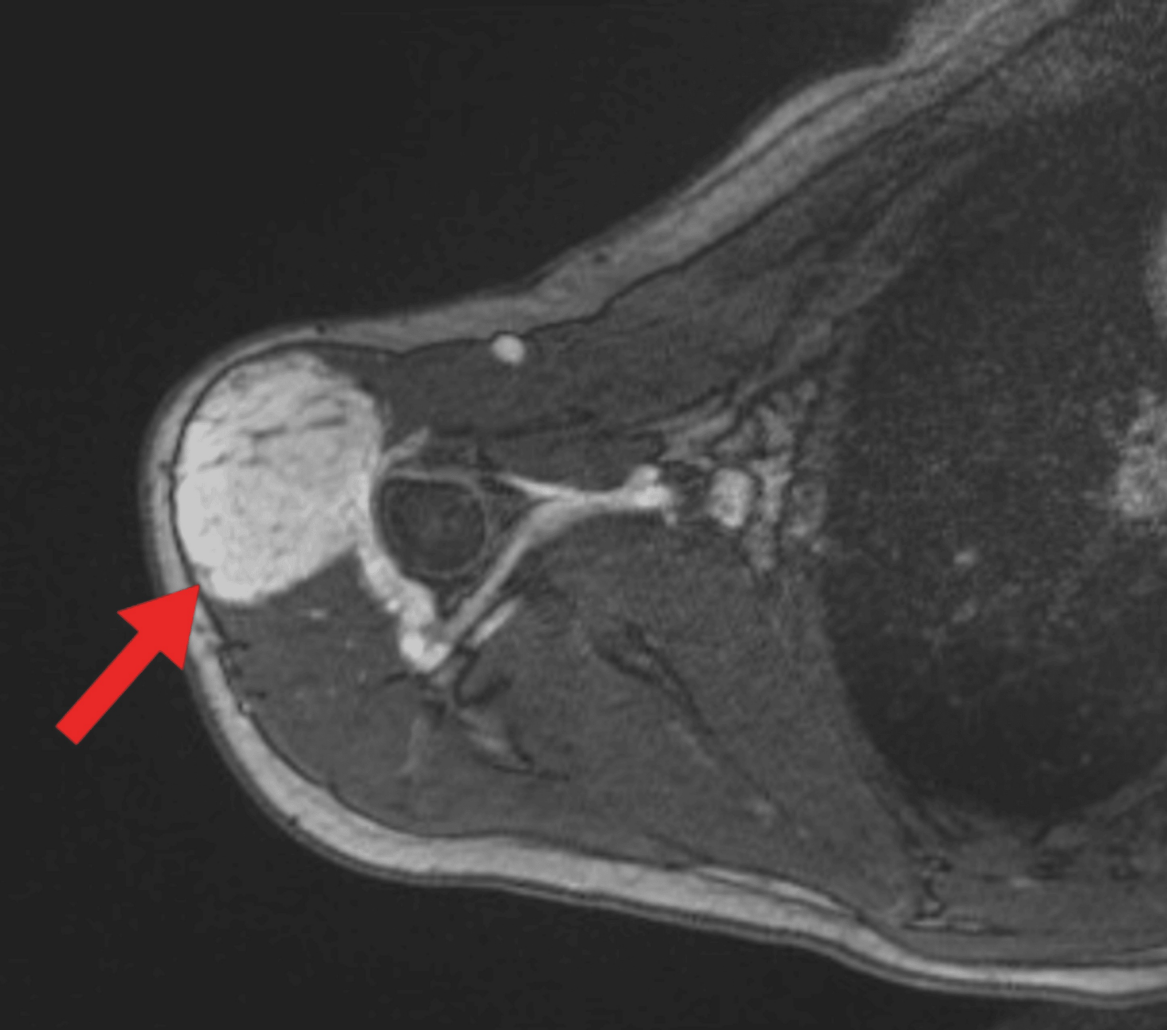
Contrast-enhanced MRI of the right humerus The red arrow points to a large fusiform area with mixed signals occupying the entire right deltoid muscle, with regions of high signal indicating fatty deposition. The lesion measured 8.5 x 4.1 x 4.7 cm, with no extension into surrounding tissues.

An MRI was done, and its results are shown in Figure 2.

**Figure 3:**
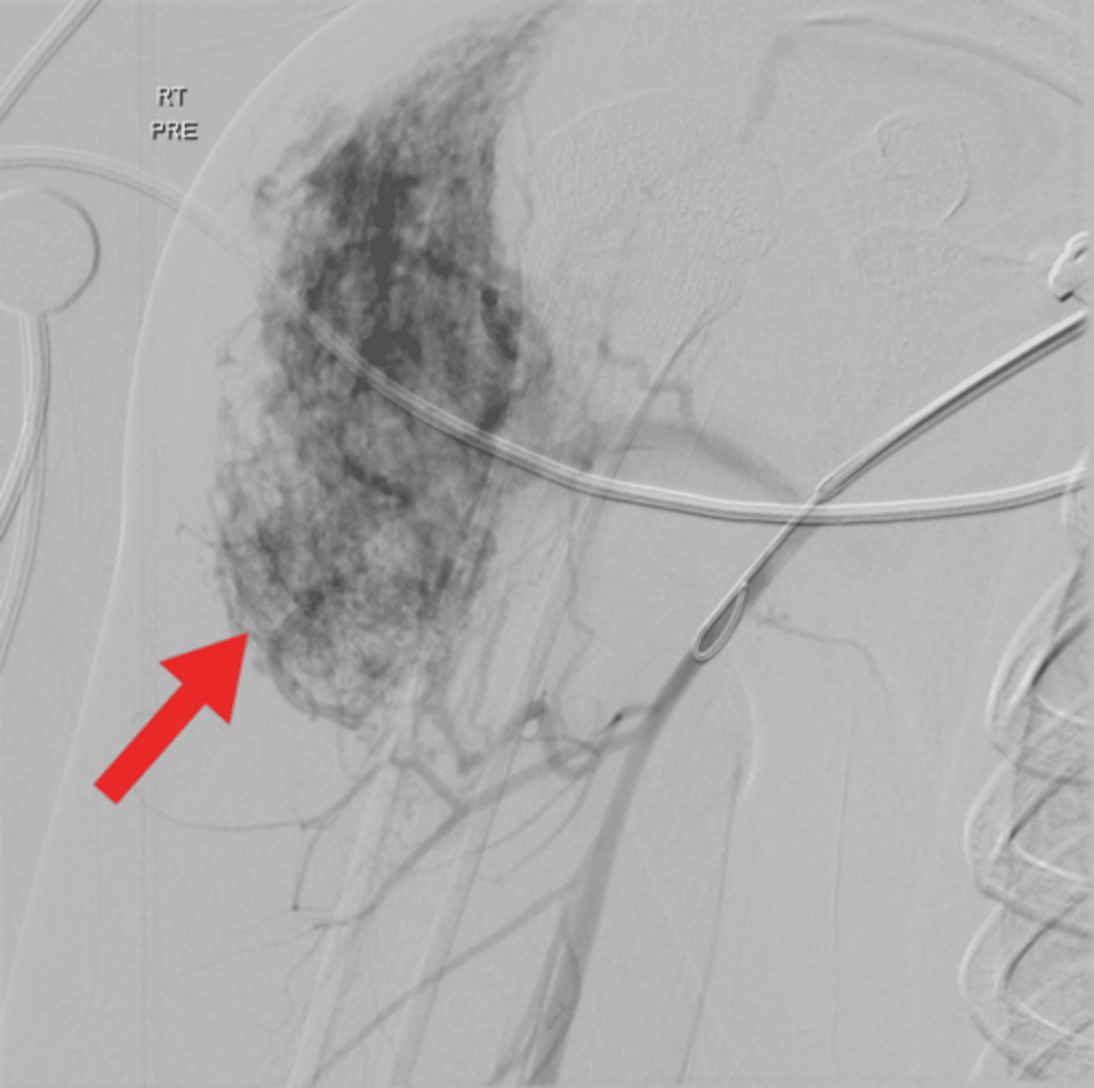
Angiography The red arrow points to the described arteriovenous malformation with four feeder vessels targeted for embolization.

Treatment began with angiography, as illustrated in Figure 3.

**Figure 4:**
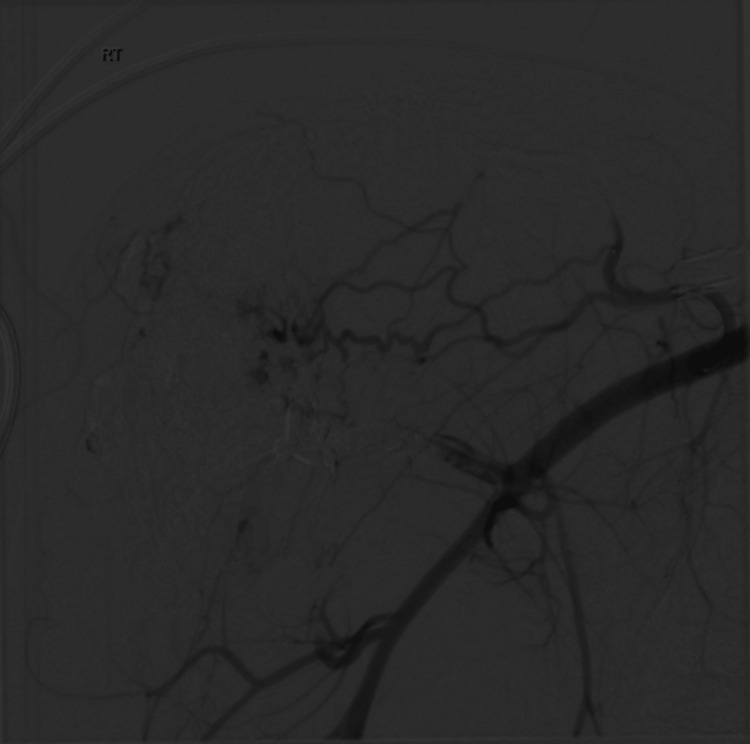
First embolization Super-selective embolization was performed using Onyx and squid chemical embolization.

Figure 4 highlights the first embolization procedure performed seven months after symptom onset, which involved super-selective embolization. The procedure was well tolerated with no immediate complications. 

**Figure 5:**
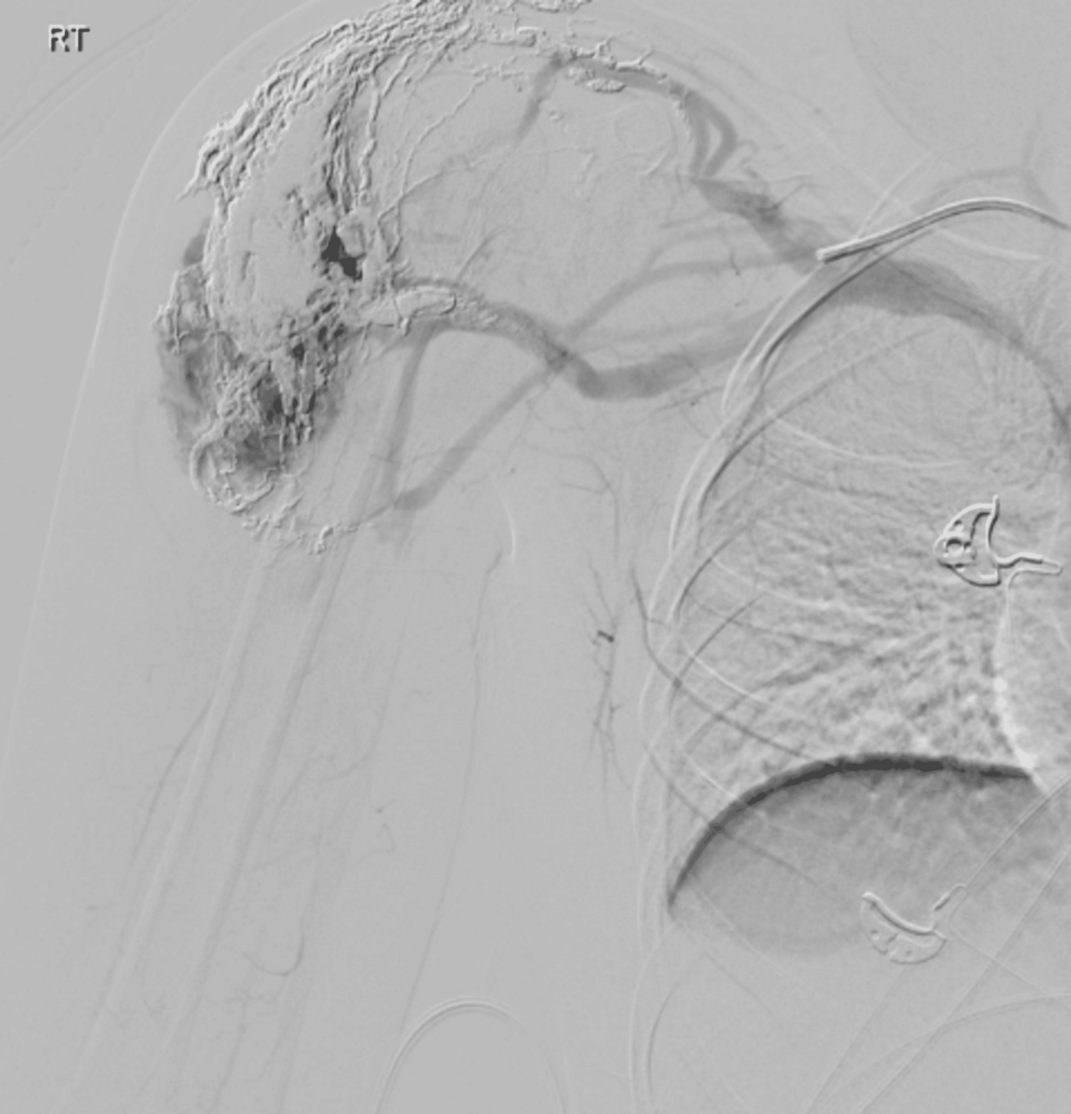
Angiography Previously injected areas with some remaining branches.

Seven months after the first embolization, recurrence was observed, accompanied by pain, particularly during shoulder movement. Angiography was done (Figure 5), which revealed previously treated areas with some remaining feeder branches.

**Figure 6:**
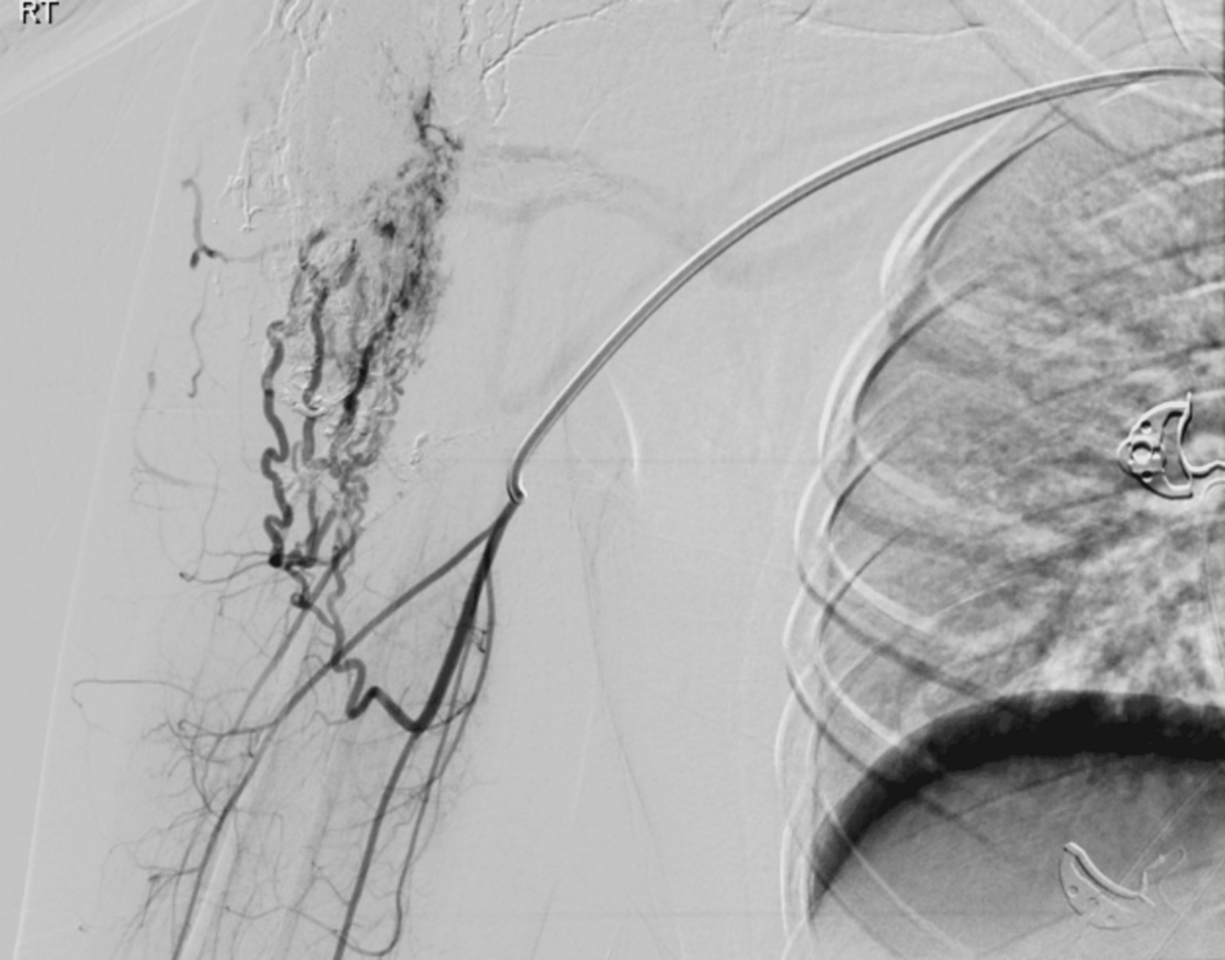
Second embolization Second embolization targeted the feeding arteries using Histoacryl and Lipiodol as embolizing agents.

A second embolization, detailed in Figure 6, was then performed, targeting the feeding arteries. This procedure resulted in a 20% reduction in the AVM size.

**Figure 7:**
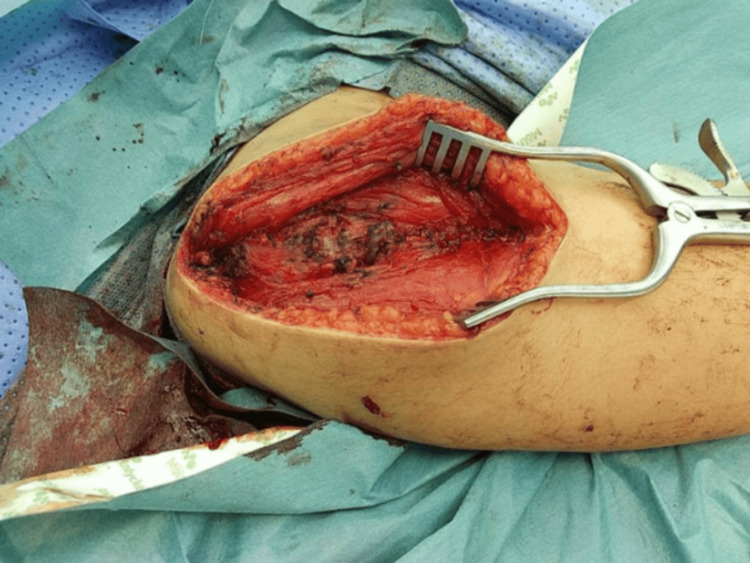
Surgical field The image shows intraoperative findings of fragile tissues and dilated vessels constituting the high-flow arteriovenous malformation in the right deltoid muscle.

Surgical resection of the AVM was performed one day after the second embolization due to persistent symptoms of pain and swelling. The surgical field is depicted in Figure 7.

**Figure 8:**
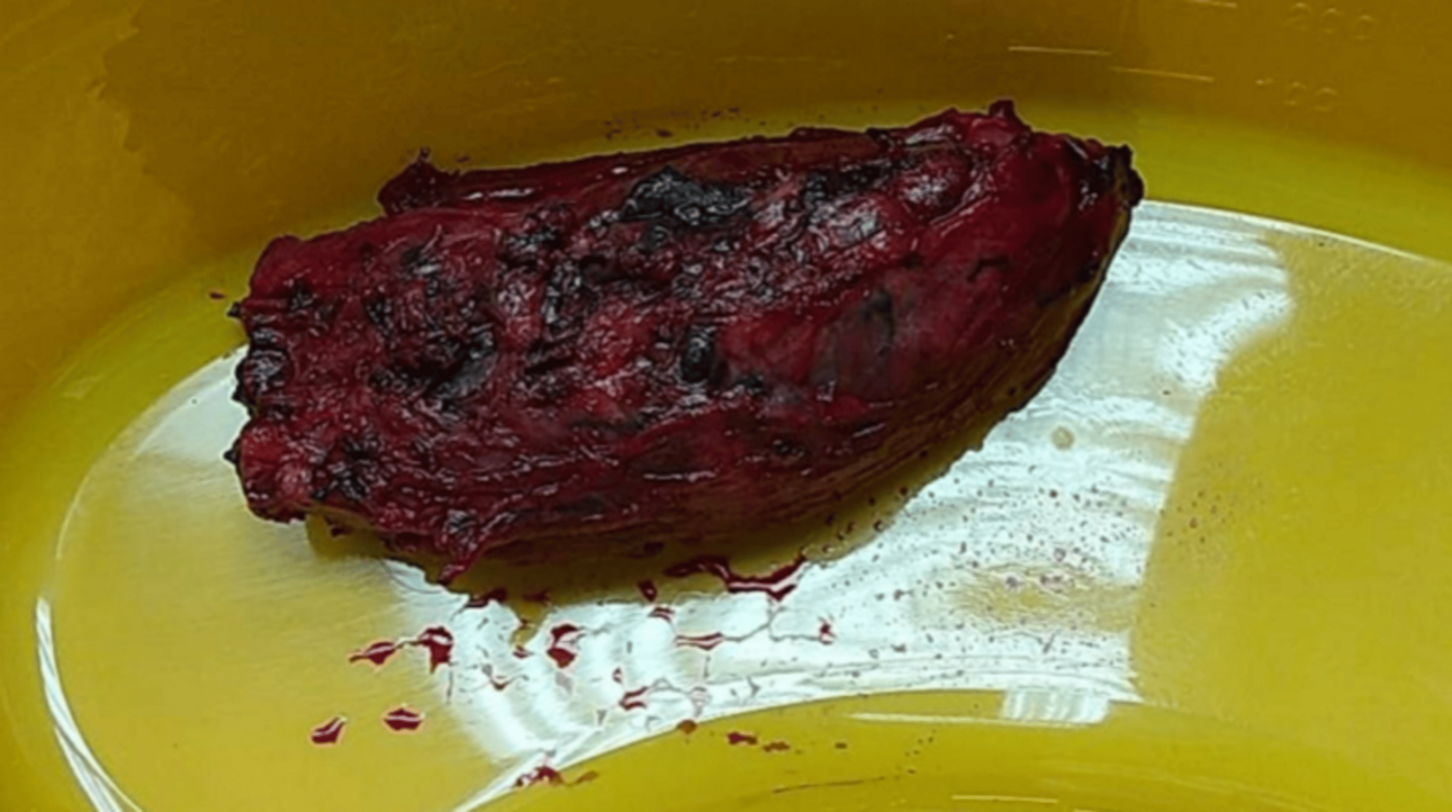
Excised specimen The image shows the excised portion of the deltoid muscle and the high-flow arteriovenous malformation.

Careful dissection was performed, and tributaries and feeding vessels were ligated. The excised specimen is depicted in Figure 8. Hemostasis was achieved, and the wound was closed without complications. 

Post-surgery, the patient experienced moderate pain (6/10) in the right shoulder, treated with diclofenac sodium, ibuprofen, and paracetamol. Over the next few days, her pain decreased to 4-5/10, with improved shoulder mobility, including intact abduction and adduction. The patient was discharged with instructions for physiotherapy evaluation for rehabilitation and a follow-up visit at the vascular clinic in two days.

The patient was seen in the vascular clinic the next week for a routine check-up and dressing change. She reported pain at the surgical site. Examination revealed an intact dressing on the right upper arm, and she was able to perform abduction and adduction of the right shoulder. The patient was advised to continue dressing changes on alternate days. An ultrasound was scheduled four weeks later to assess for any recurrence or residual AVM. The ultrasound results were reassuring. Histopathology of the excised tissue confirmed an arteriovenous malformation with no evidence of malignancy. The physiotherapist's evaluation recommended skilled physical therapy to improve joint function. Her most recent functional evaluation indicated a positive response to physiotherapy sessions.

## Discussion

AVMs are complex vascular anomalies that present significant diagnostic and therapeutic challenges. The pathophysiology of AVMs involves abnormal shunting between arteries and veins due to dysregulated angiogenesis [1]. The resulting high-flow state can cause progressive vascular dilation, tissue ischemia, and secondary complications such as pain, ulceration, and hemorrhage [2].

In managing muscle AVMs, embolization is often the first-line treatment to reduce vascular flow, minimize intraoperative bleeding, and facilitate subsequent surgical resection [3]. Studies suggest that embolization is most effective when followed by surgery within 2 to 8 weeks, as delaying beyond this period increases the risk of revascularization and recurrence [4]. In our case, two embolization procedures were required before definitive resection, highlighting the potential for persistent feeder vessels despite initial treatment.

Similar cases of AVMs in the temporalis muscle have been described in the literature. A review article on temporalis AVMs reported that these lesions can remain asymptomatic for long periods before becoming clinically evident due to hormonal changes, trauma, or increasing vascular demand [3,5]. While temporalis AVMs rarely affect muscle function, our case differs in that the deltoid AVM posed a greater risk of functional impairment, given the muscle’s role in upper limb mobility. Comparing our findings with temporalis AVMs underlines the importance of anatomical location in AVM prognosis and treatment strategies.

A key factor influencing AVM recurrence is the extent of resection and the presence of residual feeder vessels. The literature indicates that incomplete resection is associated with recurrence rates as high as 50% [6]. In our case, a thorough preoperative angiographic evaluation allowed for targeted embolization of feeder vessels, reducing intraoperative bleeding and ensuring a more complete resection.

Additionally, genetic predisposition has been implicated in AVM formation. Mutations in genes such as EPHB4 and RASA1 have been linked to hereditary vascular anomalies [7]. Future studies should investigate whether genetic screening could aid in the early identification of patients at risk for aggressive or recurrent AVMs.

A significant achievement in this case was the preservation of muscle function post-surgery. Given the essential role of the deltoid muscle in upper limb mobility, functional impairment is a major concern in AVMs located in this region. The surgical approach prioritized meticulous dissection to excise the lesion while preserving surrounding muscle fibers and neurovascular structures. The patient’s postoperative recovery, including maintaining shoulder mobility and strength, highlights the success of this approach. This underscores the importance of balancing complete resection with functional preservation in extremity AVMs to ensure optimal long-term outcomes.

The role of long-term follow-up cannot be overstated. Pediatric patients, particularly those with congenital AVMs, require lifelong monitoring due to the potential for lesion recurrence during growth spurts [8]. Imaging modalities such as Doppler ultrasound and MRI are critical tools for detecting residual or recurrent AVMs, allowing for timely intervention.

This case emphasizes the importance of a multidisciplinary approach, integrating interventional radiology, vascular surgery, and pediatric care to optimize patient outcomes. While embolization remains a cornerstone of AVM management, surgical excision is necessary in cases with persistent symptoms or high recurrence risk. Further research should explore optimal embolization timing, genetic markers for recurrence prediction, and novel therapeutic approaches, such as targeted molecular therapy for AVMs [9].

## Conclusions

This case emphasizes the importance of early diagnosis and a multidisciplinary treatment approach in pediatric AVMs. A staged approach with embolization followed by surgical resection is effective in reducing symptoms and preventing recurrence. Given the potential for lesion recurrence, long-term monitoring and follow-up imaging are essential. Future research should focus on identifying genetic markers for AVM development, optimizing embolization timing, and investigating novel treatment strategies, such as targeted molecular therapy, to further improve patient outcomes.
